# Osteopontin induces mitochondrial biogenesis in deadherent cancer cells

**DOI:** 10.18632/oncotarget.28540

**Published:** 2023-12-01

**Authors:** Gulimirerouzi Fnu, Georg F. Weber

**Affiliations:** ^1^University of Cincinnati Academic Health Center, James L. Winkle College of Pharmacy, Cincinnati, OH 45229, USA

**Keywords:** metastasis, metabolism, anchorage independence, mitochondrial mass, peroxide

## Abstract

Metastasizing cells display a unique metabolism, which is very different from the Warburg effect that arises in primary tumors. Over short time frames, oxidative phosphorylation and ATP generation are prominent. Over longer time frames, mitochondrial biogenesis becomes a pronounced feature and aids metastatic success. It has not been known whether or how these two phenomena are connected. We hypothesized that Osteopontin splice variants, which synergize to increase ATP levels in deadherent cells, also increase the mitochondrial mass via the same signaling mechanisms. Here, we report that autocrine Osteopontin does indeed stimulate an increase in mitochondrial size, with the splice variant -c being more effective than the full-length form -a. Osteopontin-c achieves this via its receptor CD44v, jointly with the upregulation and co-ligation of the chloride-dependent cystine-glutamate transporter SLC7A11. The signaling proceeds through activation of the known mitochondrial biogenesis inducer PGC-1 (which acts as a transcription coactivator). Peroxide is an important intermediate in this cascade, but surprisingly acts upstream of PGC-1 and is likely produced as a consequence of SLC7A11 recruitment and activation. *In vivo*, suppression of the biogenesis-inducing mechanisms leads to a reduction in disseminated tumor mass. This study confirms a functional connection between the short-term oxidative metabolism and the longer-term mitochondrial biogenesis in cancer metastasis – both are induced by Osteopontin-c. The results imply possible mechanisms and targets for treating cancer metastasis.

## INTRODUCTION

The metabolism in metastasizing cells is dramatically different from the Warburg effect. It is associated with peroxide production and mitochondrial activation, leading to increased ATP generation [1]. Through implementing this energy uptick, which non-transformed epithelial cells are incapable of, deadherent cancer cells achieve anti-anoikis. Over a longer timeframe (metastasizing cells linger in the vasculature for weeks [2]), mitochondrial biogenesis has also been observed [3, 4], but it has not been clear whether the functional activation of ATP production and the structural adaptation of mitochondrial biogenesis are downstream effects of the same signaling mechanisms or are two distinct manifestations.

The detachment of healthy epithelial cells causes anoikis due to an impairment of glucose uptake and energy production [5]. The cytokine Osteopontin (OPN) mediates tumor progression, in part through fostering anti-anoikis and expansion under deadherent conditions [6]. In transformed epithelial cells, including breast cancer, Osteopontin splice variants support anchorage-independence via inducing the energy metabolism. Specifically, Osteopontin-a signaling elevates the cellular glucose levels [7], while signaling from Osteopontin-c upregulates glutamine and glutamate, which can feed into the tricarboxylic acid cycle and can upregulate creatine. Consecutively, the cellular ATP levels are elevated [1]. These results are supported by multiple reports, which have shown that some of the metabolites, which our laboratory has found to be upregulated by Osteopontin (including serine, glycine and glycerol), are typically elevated in aggressive cancers [8–10]. Therefore, the autocrine effects of Osteopontin variants are instrumental in the anchorage-independent survival of disseminating malignant cells. While Osteopontin-a increases glucose uptake, Osteopontin-c utilizes this glucose in the hexose monophosphate shunt and tricarboxylic acid cycle to generate increased amounts of ATP [11]. Whether Osteopontin is also a contributor to mitochondrial biogenesis has been unexplored.

We have analyzed profiles of gene expression in breast cancer metastases and metastases in general, reported in the GEO (Gene Expression Omnibus) database, which has allowed us to identify gene regulation changes in disseminated growths relative to their target site or to their tumor of origin [12]. There is overlap between the gene expression patterns that characterize metastases and the genes induced in cancer cells by Osteopontin under deadhesion (gene expression analyses from soft agar experiments). The master regulator of mitochondrial biogenesis, PPARG, is upregulated in both settings, in deadherent cells under the effect of Osteopontin as well as in breast cancer metastases to multiple sites.

Invasive cancer cells use the transcription coactivator peroxisome proliferator-activated receptor γ, coactivator 1α (PPARGC1A, PGC-1α) to enhance the oxygen consumption rate, oxidative phosphorylation, and mitochondrial biogenesis. Silencing of PGC-1α in cancer cells suspends their invasive potential and attenuates metastasis without affecting proliferation or primary tumor growth [3]. The PGC-1 family of regulated transcriptional coactivators (consisting of PGC-1α, PGC-1β and PRC) plays a central role in a regulatory network that governs the control of respiratory function and mitochondrial biogenesis. These coactivators target multiple transcription factors, including the nuclear respiratory factors NRF1, NRF2, and the orphan nuclear hormone receptor ERRα [13]. Another player in this regulatory chain is the transcription factor BACH1 (BTB and CNC homology 1). Like NRF2, BACH1 belongs to the cap’n’collar b-Zip family of proteins that bind to antioxidant response elements consecutive to changes in redox states. Under conditions of oxidative stress, the oxidation of heme-containing proteins releases free heme, which stimulates the degradation of BACH1. By contrast, in low-oxidative stress conditions, heme levels are low, which leads to BACH1 stabilization. BACH1 may displace NRF2 from antioxidant response elements and act primarily as a transcriptional repressor for antioxidant genes. BACH1 is contained in the metastatic signatures by various cancers [14]. How these intermediates, individually or jointly, are engaged downstream of Osteopontin remains to be analyzed.

Here, we study the induction of mitochondrial biogenesis by Osteopontin variants in deadherent breast tumor cells. The mechanistic elucidation will enhance our understanding of metastasis formation and may point the way toward novel treatment modalities.

## RESULTS

### Osteopontin splice variants support mitochondrial biogenesis

**Figure 1:**
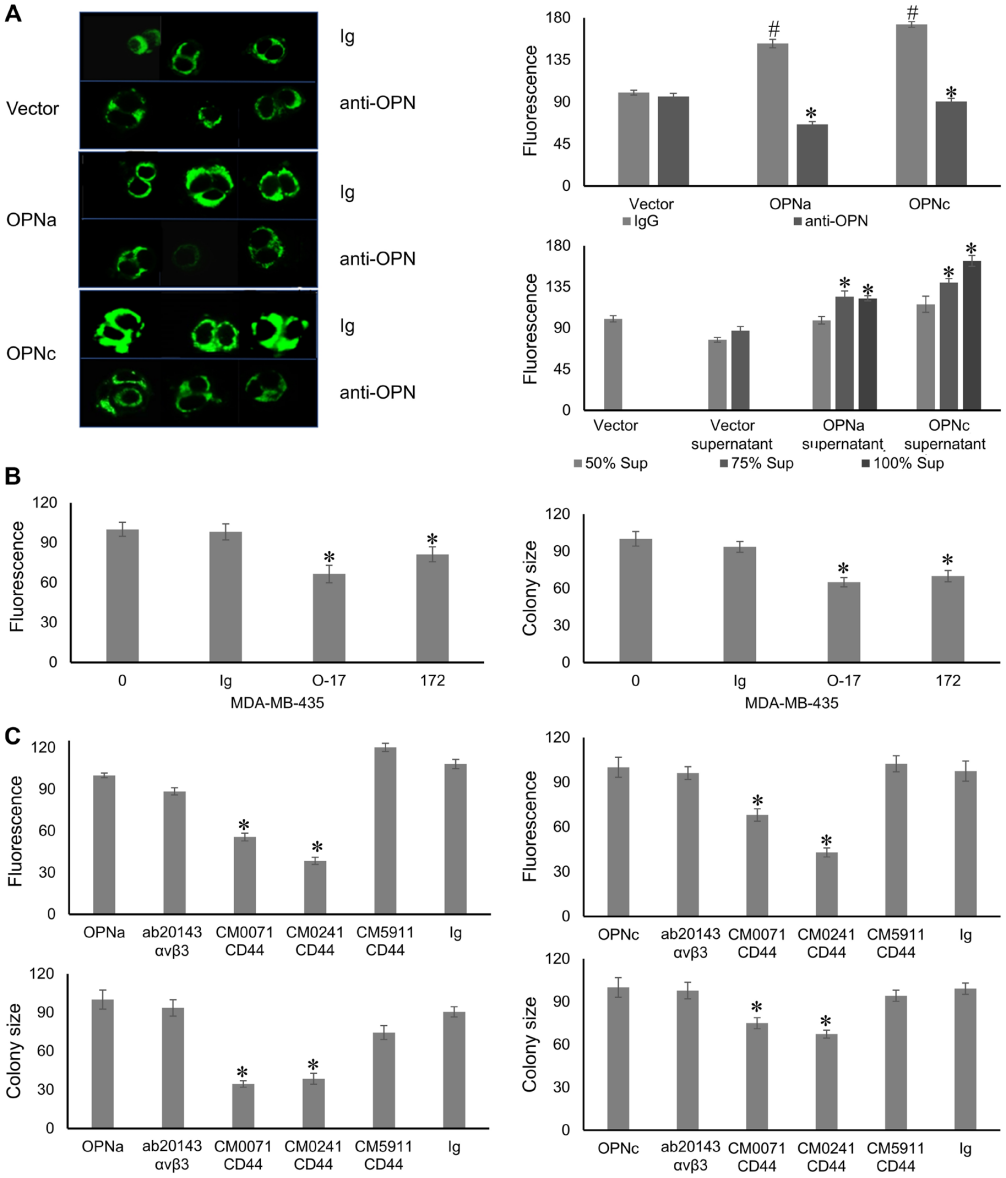
Autocrine OPN effectuates mitochondrial biogenesis via its receptor CD44v. (**A**) OPN is necessary and sufficient for mitochondrial biogenesis in soft agar colonies. (left and top right, measured as area of fluorescent signal) Transfection with OPNc strongly induced mitochondrial biogenesis as compared to vector. OPNa had an intermediate effect. Addition of anti-OPN antibody O-17 but not control Ig to deadherent cultures of MCF-7 transfectants every other day suppressed the mitochondrial size. (bottom right) Addition of 50%, 75%, 100% supernatants from vector, OPNa or OPNc to deadherent MCF-7 cultures increased the mitochondrial size, with OPNc having a stronger effect than OPNa. (**B**) Endogenous OPN mediates mitochondrial biogenesis. MDA-MB-435 cells were plated in soft agar. Every other day, they were treated with anti-OPN antibody O-17, anti-OPNc antibody clone 172, control Ig or left untreated (0). Measurements were done either after 5 days for mitochondrial fluorescence (left panel) or after 14 days for colony size (right panel). Fluorescent signal and colony size are expressed as relative units (control = 100 units). Representative photographs are shown in Supplementary Figure 1. (**C**) The OPN effect on mitochondria and on colony formation is exerted through CD44v. Antibodies to the CD44 variable domain, but not to the far N-terminus of CD44 or to Integrin α_v_β_3_, reversed the increase of mitochondrial size (top panels) in soft agar as well as soft agar colony formation (bottom panels) by OPNa-transfected (left bar graphs) and OPNc-transfected (right bar graphs) MCF-7 cells. ^#^ = significant increase compared to vector control; ^*^ = significant change by treatment (*p* < 0.05). Fluorescent intensity and colony size are expressed as relative units (control = 100 units). Abbreviation: Sup: supernatant. Representative photographs are shown in Supplementary Figure 2.

Using the OPNa or OPNc transfectants (or vector control) of the non-invasive MCF-7 breast tumor cells, we visualized the sizes of fluorescently labeled mitochondria (as a readout for mitochondrial mass, which is indicative of biogenesis) after 5 days of soft agar growth. Corresponding to the strong (OPNc) or intermediate (OPNa) induction of anchorage-independent growth (as reported before [6]), transfected OPNc significantly induced mitochondrial biogenesis, while transfected OPNa had an intermediate effect compared with the vector control (Figure 1A).

Because OPN can also be intracellular [15], we studied whether the OPN effect on mitochondrial size requires its secretion. The addition of anti-OPN antibody, but not control immunoglobulin, to the soft agar cultures of the transfectants – every other day with the supplementation of medium – suppressed the OPN-dependent mitochondrial biogenesis. Conversely, the addition – every second day – of transfectant supernatants (from MCF-7 vector, MCF-7 OPNa, or MCF-7 OPNc) to soft agar plates containing parent MCF-7 cells induced mitochondrial biogenesis in proportion to the OPN variant present (Figure 1A). MDA-MB-435 cells, although of debated lineage origin, are malignant and strongly secrete the splice variants -a, -b, and -c. The MDA-MB-435 mitochondrial size (according to fluorescence measurement) as well as soft agar colony formation were inhibitable by antibody O-17 (to pan-OPN) and by antibody clone 172 (a rabbit monoclonal antibody to OPNc), but not by a control Ig (Figure 1B). Hence, OPN secretion is necessary and sufficient for mitochondrial biogenesis.

### The autocrine Osteopontin effect on mitochondria is exerted via CD44v

To assess how secreted OPN forms induce mitochondrial biogenesis, we blocked the main OPN cell surface receptors with neutralizing antibodies (compared to Ig controls). MCF-7 cells express CD44 and Integrin α_v_β_3_ [16, 17]. An anti-Integrin α_v_β_3_ antibody did not impact either mitochondrial size or soft agar colony formation. An antibody to the far N-terminus of CD44 (CM5911, binds amino acids 202–217 in UniProt P16070-1, targeting the heparin binding site, which does not overlap with the OPN binding site) had no or marginal effect, whereas antibodies to the CD44 variable domain (CM0071 and CM0241, do not bind the N-terminal amino acids 1-300 in CD44, UniProt P16070-1) suppressed the increase in mitochondrial size as well as the formation of soft agar colonies (Figure 1C). Therefore, the autocrine OPN-dependent mitochondrial biogenesis is mediated via binding of the secreted cytokine to its receptor CD44v.

### Osteopontin signaling proceeds via PGC-1

**Figure 2:**
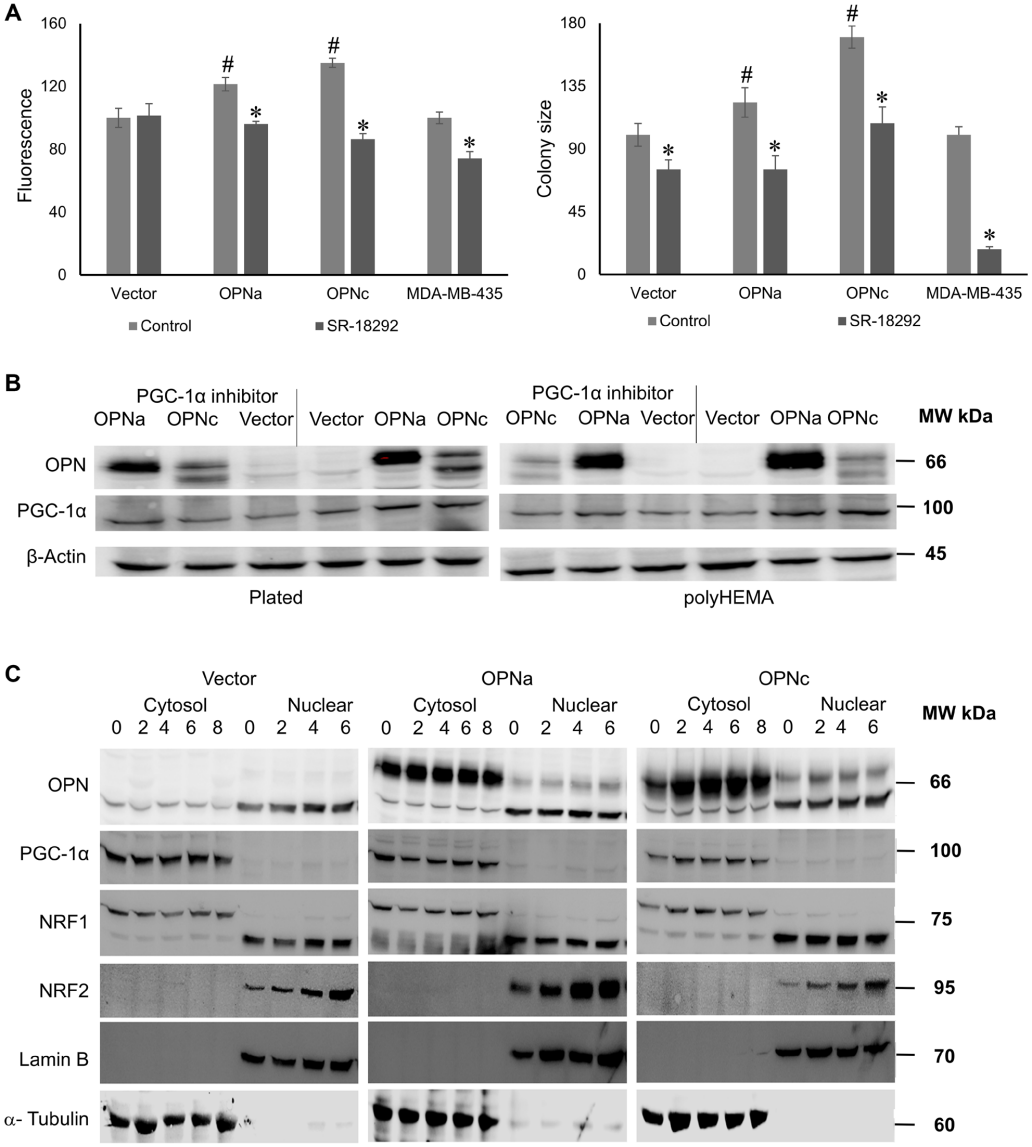
PGC-1α signaling is an essential mediator of the OPN effect on mitochondria. (**A**) Measurements of mitochondrial size (as fluorescent area, left) and colony formation (right) by MCF-7 transfectants in the presence or absence of the PGC-1α inhibitor SR-18292. ^#^ = significant increase compared to vector control; ^*^ = significant change by treatment (*p* < 0.05). Fluorescent intensity and colony size are expressed as relative units (control = 100 units). Representative photographs are shown in Supplementary Figure 2. (**B**) Western blot for OPN and PGC-1α expression levels in plated (left) or poly-HEMA (right) growth conditions and in the presence or absence of the PGC-1α inhibitor SR-18292 over 48 hours. β-Actin served as a loading control. (**C**) Western blot of OPN together with PGC-1α and its downstream targets NRF1 and NRF2 at various time points under deadherent conditions on poly-HEMA (0 hours is plated on plastic). The cytosolic and nuclear fractions were analyzed to assess nuclear translocation. Lamin B served as a nuclear marker, while α-Tubulin provided a cytosolic marker.

To gain mechanistic insight, we followed up on prior reports that invasive cancer cells use the transcriptional coactivator PPARGC1A (PGC-1α) to enhance oxidative phosphorylation and mitochondrial biogenesis [3]. We tested the effects of chemical PGC-1α modulators on the OPN-reliant anchorage-independence. SR-18292 inhibits PGC-1α gluconeogenic activity and reduces the co-activation of HNF4α by modulating the interaction between GCN5 and PGC-1α. We treated the MCF-7 transfectants in soft agar with 20 μM SR-18292 every other day up to 5 days, then observed MitoTracker fluorescence under a microscope and measured the areas. The chemical suppression of PGC-1α reduced the mitochondrial size of OPNa transfectants and OPNc transfectants after 5 days and the soft agar colony size after 11 days. In colony formation, the vector-transfected cells were also inhibited by SR-18292, which suggests a fundamental role for PGC-1 in supporting anchorage-independence over extended time periods. Further, the endogenously OPN-splice-variant-producing MDA-MB-435 cells likewise were sensitive to SR-18292 (Figure 2A). We also added 20 μM SR-18292 to the transfectants (plated on conventional dishes or on poly-HEMA-coated plates over 48 hours) for Western blotting to detect OPN, PGC-1α, and β-Actin. Expectedly, treating the MCF-7 cells with the PGC-1α inhibitor SR-18292 did not affect the PGC-1α expression levels compared to untreated controls (Figure 2B). Thus, PGC-1α activation is a necessary intermediate in the OPN effect on mitochondrial biogenesis. ZLN005 is a tissue-specific PGC-1α transcriptional activator. To study whether PGC-1α is also sufficient for mitochondrial biogenesis, we added 20 μM ZLN005 to the plated MCF-7 cells over 48 hours. Then, we tested by Western blotting the expression levels of OPN, PGC-1α, and β-Actin. Treating MCF-7 vector with ZLN005 did not affect the PGC-1α expression level compared to untreated vector transfectants as a control. This result indicated that this tissue-specific activator may not work in breast tumor cells (Supplementary Figure 3).

The transcription factors NRF1 and NRF2 have been described as downstream effectors of PGC-1 [4]. They have been reported to translocate upon activation from the cytoplasm to the nucleus. We plated 3 × 10^5^ cells/well in 6-well plates under deadherent conditions over 2–8 hours (with cells harvested from plated conditions representing time point 0). We prepared cytosolic and nuclear extracts and separated them on SDS-PAGE gels to perform Western blotting. The membranes were probed for pan-OPN, PGC-1α, NRF1, NRF2 as well as the cytoplasmic and nuclear marker proteins α-Tubulin and Lamin B. PGC-1α remained cytosolic and did not change over time. NRF1 was present in both fractions, also without substantial change over time. However, NRF2 was entirely nuclear and its signal increased with prolonged deadhesion. Unexpectedly, there were no discernible differences among the transfectants OPNc, OPNa and vector (Figure 2C). An increase of nuclear NRF2 appears to be required for deadherent survival in all cells.

To test the relevance of the cell line data for *in situ* tumors by patients, we reanalyzed profiles of gene expression in metastases from public information (breast cancer metastases and metastases in general, deposited in GEO), which we reported on previously [12]. The master regulator of mitochondrial biogenesis, PPARG, as well as PPARGC1A and the downstream effectors and transcription factors NRF1 [4] and BACH1 [14], are upregulated in various metastases as compared to the primary tumor or to the target site. Likewise, SLC7A11 (see below) is often overexpressed (Table 1).

**Table 1:** Gene expression pertinent to mitochondrial biogenesis in human metastases recorded in GEO

	Peritoneum			Liver			Bone			Adrenal		
Gene symbol	logFC	SD	FDR	logFC	SD	FDR	logFC	SD	FDR	logFC	SD	FDR
SLC7A11							0.22699	0.10909	4.6E-05			
PPARG	0.46515	0.1006	1.4E-12									
PPARGC1A	0.627	0.0845	1.4E-12	0.29468	0.17114	2.1E-09						
NRF1	0.90534	0.0828	2.4E-21	0.54592	0.10218	9.5E-11				0.64905	0.02899	1.8E-05
BACH1							0.31497	0.18634	2.2E-05			
	**Breast**			**Kidney**			**Prostate**					
**Gene symbol**	**logFC**	**SD**	**FDR**	**logFC**	**SD**	**FDR**	**logFC**	**SD**	**FDR**			
SLC7A11	0.11891	0.01956	5.4E-20	0.06354	0.20522	0.02854						
PPARG	0.02657	0.0761	2.8E-05				0.1942	0.06662	0.00152			
PPARGC1A	1.4E-01	0.09991	7E-36									
NRF1	0.13882	0.12917	5.2E-12									
BACH1							0.08529	0.06315	0.00922			

Analyzed were metastasis signatures in GEO for relevant pathway genes significantly upregulated in the metastases over host tissue and genes significantly upregulated in the metastases compared to the primary tumors [12]. Abbreviations: Upper portion: comparison to metastatic target organs; lower portion: comparison to primary tumors; logFC: logarithm of fold change; SD: standard deviation; FDR: false discovery rate.

### Reactive oxygen species are essential intermediaries of the Osteopontin effect

**Figure 3:**
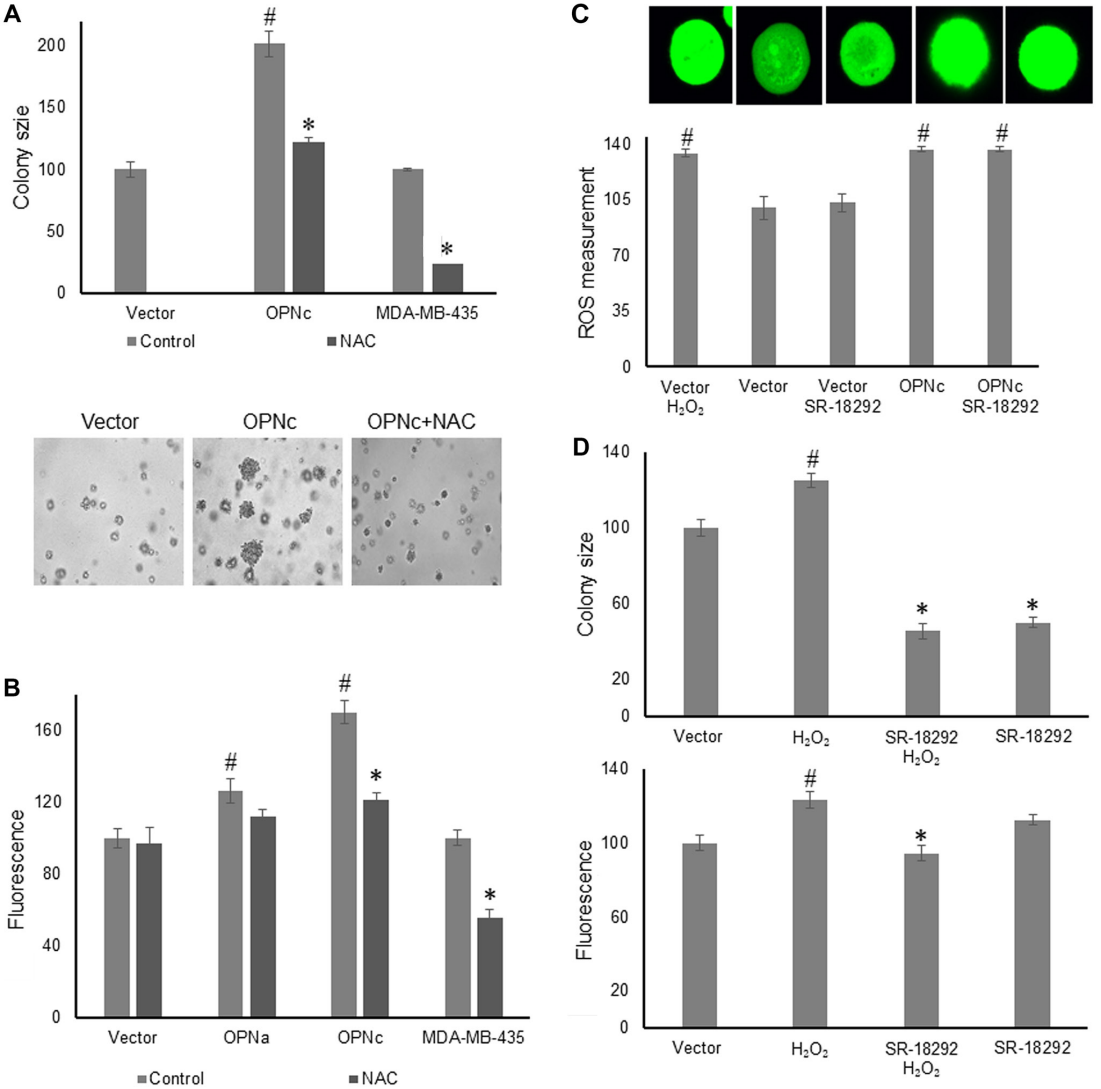
Peroxide is an essential intermediary of the osteopontin effect. (**A**) Treatment of deadherent MCF-7 cells, MCF-7 OPNc cells, and MDA-MB-435 cells with 2 mM NAC over 11 days in soft agar, followed by the assessment of colony size. The lower panel shows representative photographs for the MCF-7 cells. (**B**) Treatment of MCF-7 transfected cells (vector, OPNa, OPNc) as well as MDA-MB-435 cells in soft agar with 2 mM NAC for 5 days before assessment of mitochondria size (relative units). (**C**) MCF-7 vector and MCF-7 OPNc were grown in soft agar for 16 hours. Measurement of intracellular reactive oxygen species according to di(acetoxymethyl ester)(6-carboxy-2’,7’-dichlorodihydrofluorescein diacetate) fluorescence. The upper panel displays representative microscopy pictures. Fluorescent intensity and colony size are expressed as relative units (control = 100 units). (**D**) Treatment of deadherent MCF-7 vector cells with 10 μM H_2_O_2_, 10 μM of the PGC-1α inhibitor SR-18292, or both before assessment of colony size (upper graph, 11 days) and mitochondria size (lower graph, 5 days). Representative photographs are shown in Supplementary Figure 4.

Peroxides have been widely described to mediate anchorage independence (discussed in [11]). Because the peroxide scavenger Glutathione Peroxidase (GPX) plays an important regulatory role in this process, we evaluated the effect on mitochondrial biogenesis by adding the glutathione precursor N-acetyl L-cysteine (NAC). As had been reported previously [1], NAC at 2 mM every other day suppressed the formation of soft agar colonies by MCF-7 OPNc after 11 days (Figure 3A). We therefore treated MCF-7 cells, transfected with vector, OPNa, or OPNc in soft agar with 2 mM NAC and measured the size of the fluorescently labeled mitochondria after 5 days. The presence of NAC was sufficient for suppressing the mitochondrial size induced by OPNc, but not by OPNa or vector (Figure 3B). We also treated MDA-MB-435 cells, which strongly express three spliced forms of OPN (-a, -b, and -c), with NAC under the same conditions as the MCF-7 transfectants, and found a similar effect (Figure 3A, 3B).

It was important to assess whether peroxides act upstream or downstream of PGC-1. We plated MCF-7 vector cells in soft agar and treated them with hydrogen peroxide alone, hydrogen peroxide plus the PGC-1α inhibitor SR-18292, SR-18292 alone, and culture medium as a control. Then, we measured either colony size after 11 days or mitochondria size after 5 days. We reasoned, if hydrogen peroxide acts upstream of PGC-1, then the induction of mitochondrial biogenesis by the peroxide will be reversible with the PGC-1 inhibitor. Conversely, if PGC-1 is upstream of the site of peroxide action, then hydrogen peroxide will exert its effect regardless of the presence or absence of the inhibitor. SR-18292 did reverse the peroxide-induced mitochondrial biogenesis as well as colony size, indicating that the peroxides that are important for anchorage-independent growth are generated and act upstream of PGC-1 (Figure 3C, Supplementary Figure 4). Of note, as was observed in Figure 2, while the mitochondria size by MCF-7 vector was unaffected by SR-18292, the soft agar colony size (measured after a much longer time period) was reduced by the inhibitor.

We further corroborated the sequence of the peroxide effect and the PGC-1 action by measuring the intracellular reactive oxygen species with the fluorescent dye di(acetoxymethyl ester)(6-carboxy-2’,7’-dichlorodihydrofluorescein diacetate) after 16 hours in soft agar. The fluorescence in vector transfectants was moderate and remained unchanged by the presence of SR-18292. However, the addition of H_2_O_2_ increased the fluorescent signal. The MCF-7 OPNc transfectants displayed a strong signal, roughly comparable to vector plus H_2_O_2_. This was not inhibitable by SR-18292 (Figure 3D). Again, the evidence supported the conclusion that peroxides act upstream of PGC-1.

### The channel SLC7A11 is a candidate redox mediator

Previous reports on redox signaling in metastasis have mostly assumed that the mitochondria serve as the source of the peroxides generated. Mitochondrial uncoupling (a dissociation between mitochondrial membrane potential generation and its use for ATP synthesis) has been put forth as a likely mechanism. Our unexpected results of an upstream point of action mandated a search for a likely redox generator.

**Figure 4:**
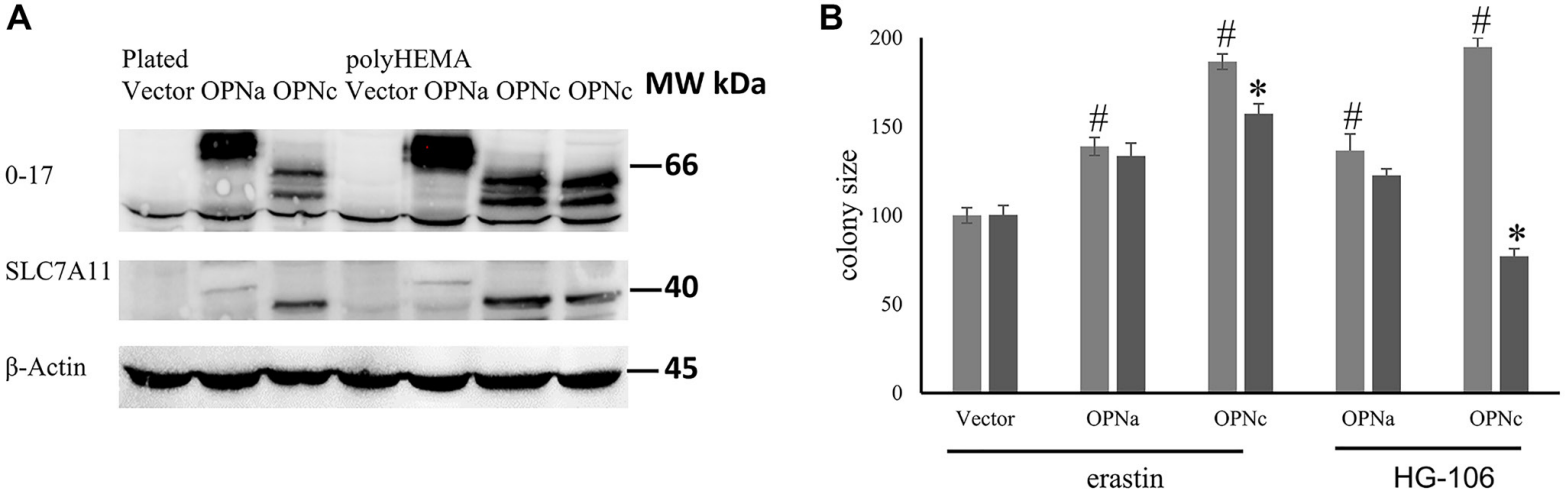
Involvement of SLC7A11. (**A**) Western blot confirming the expression of OPN (antibody O-17) and SLC7A11 in transfected MCF-7 cells (plated on plastic or on poly-HEMA) as compared to Actin control. (**B**) The SLC7A11 inhibitors erastin (0.75 μM) or HG106 (25 μg/ml) were added to soft agar colonies of MCF-7 vector, MCF-7-OPNa and MCF-7-OPNc every other day for 16 days. Then, the colony sizes were measured. ^#^ = significant change compared to vector control; ^*^ = significant change by treatment (*p* < 0.05). Colony size is expressed as relative units (control = 100 units).

Here, we have found the OPN-dependent mitochondrial biogenesis to require the engagement of CD44v (see Figure 1). CD44 can associate with the chloride-dependent cystine-glutamate transporter SLC7A11 (sometimes referred to as system Xc- or xCT). Also, OPNc was previously described to induce glutamate in a manner that is reversible by NAC [1]. This raised the possibility that OPNc ligates CD44v, which then recruits and activates SLC7A11. In UniProt, one of the GO categories associated with SLC7A11 is “GO:0051775 response to redox state” (https://www.uniprot.org/uniprotkb/Q9UPY5/entry). When testing for the expression of SLC7A11 in MCF-7 cells, we found that Western blotting showed no signal for this channel in vector-transfected or OPNa-transfected MCF-7 cells. However, the transfection of OPNc into MCF-7 cells led to a substantial upregulation and conspicuous expression of SLC7A11 in two distinct clones (Figure 4A). This is in line with precedent that ligands can induce the expression of their binding partners. As a case in point, Osteopontin induces the expression of CD44 [18]. Consistently, the chemical SLC7A11 inhibitors erastin as well as HG106 at low concentrations [19, 20] suppressed the soft agar colony formation by MCF-7 OPNc cells but not by MCF-7 OPNa or vector control (Figure 4B).

### The mediators of mitochondrial biogenesis are required for disseminated growth *in vivo*

**Figure 5:**
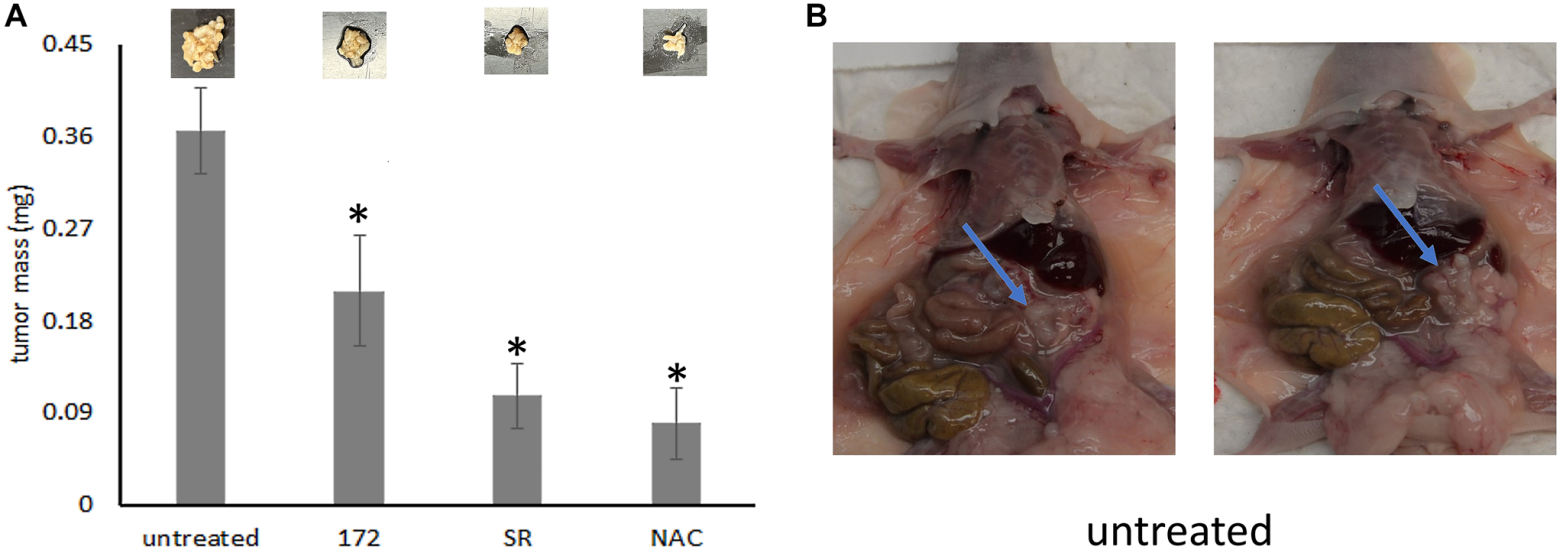
The mediators of mitochondrial biogenesis are required for cancer spread. Balb^nu/nu^ mice received i.p., injection of 1 × 10^6^ MDA-MB-435 cells. Drug treatment was administered i.p., every other day (doses: NAC 100 mg/kg, SR-18292 45 mg/kg) in a total volume of 200 μl PBS. Antibody treatment was given every 4 days at 60 μg in 200 μl PBS. The experiment was terminated on day 21. (**A**) The bar graph displays the mass of the tumors (mg) in the abdominal cavity. Above the bars are pictures of one representative tumor each. (**B**) *In situ* tumors in two representative untreated mice. For all bar graphs, ^*^ = significant change by treatment (*p* < 0.05). The error bars indicate standard error of the mean.

Three weeks after intraperitoneal injection of MDA-MB-435 cells, the tumor burden without treatment (sham injections of PBS) reached about 360 mg. When treated with rabbit monoclonal anti-hOPNc clone 172 (every 4 days at 60 μg in 200 μl PBS), with NAC (100 mg/kg every other day), or with the PGC-1 inhibitor SR-18292 (45 mg/kg every other day), the total mass of tumor growths retrieved from the abdominal cavity was significantly reduced in each case (Figure 5). Of note, the incomplete reduction in tumor growth by antibody clone 172 is likely due to suboptimal dosing. In murine models, 100–200 μg every other day are commonly used. Due to limited reagent availability, we applied 60 μg every 4 days.

### STAT3, a downstream target for OPNa signaling, is not involved in mitochondrial biogenesis

Osteopontin-a upregulates the levels of glucose in breast cancer cells through STAT3, likely via its transcriptional targets apolipoprotein D and IGFBP5 [7]. Therefore, we asked whether STAT3 may be involved in mediating mitochondrial biogenesis. We utilized double-transfectant MCF-7 cells expressing OPNa or OPNc in conjunction with vector or dominant negative STAT3. Consistent with the previous report, dominant-negative STAT3 (dnSTAT3) selectively reduced the soft agar colony formation by OPNa, but not by OPNc. However, dnSTAT3 did not compromise the mitochondrial size in deadherent MCF-7 transfectants (Supplementary Figure 5). Whereas STAT3 contributes to the OPNa-dependent glucose elevation in deadherent cells (and thus contributes to increased soft agar colony size), it is not involved in the pathway to mitochondrial biogenesis, which is mediated by peroxides and PGC-1.

## DISCUSSION

**Figure 6:**
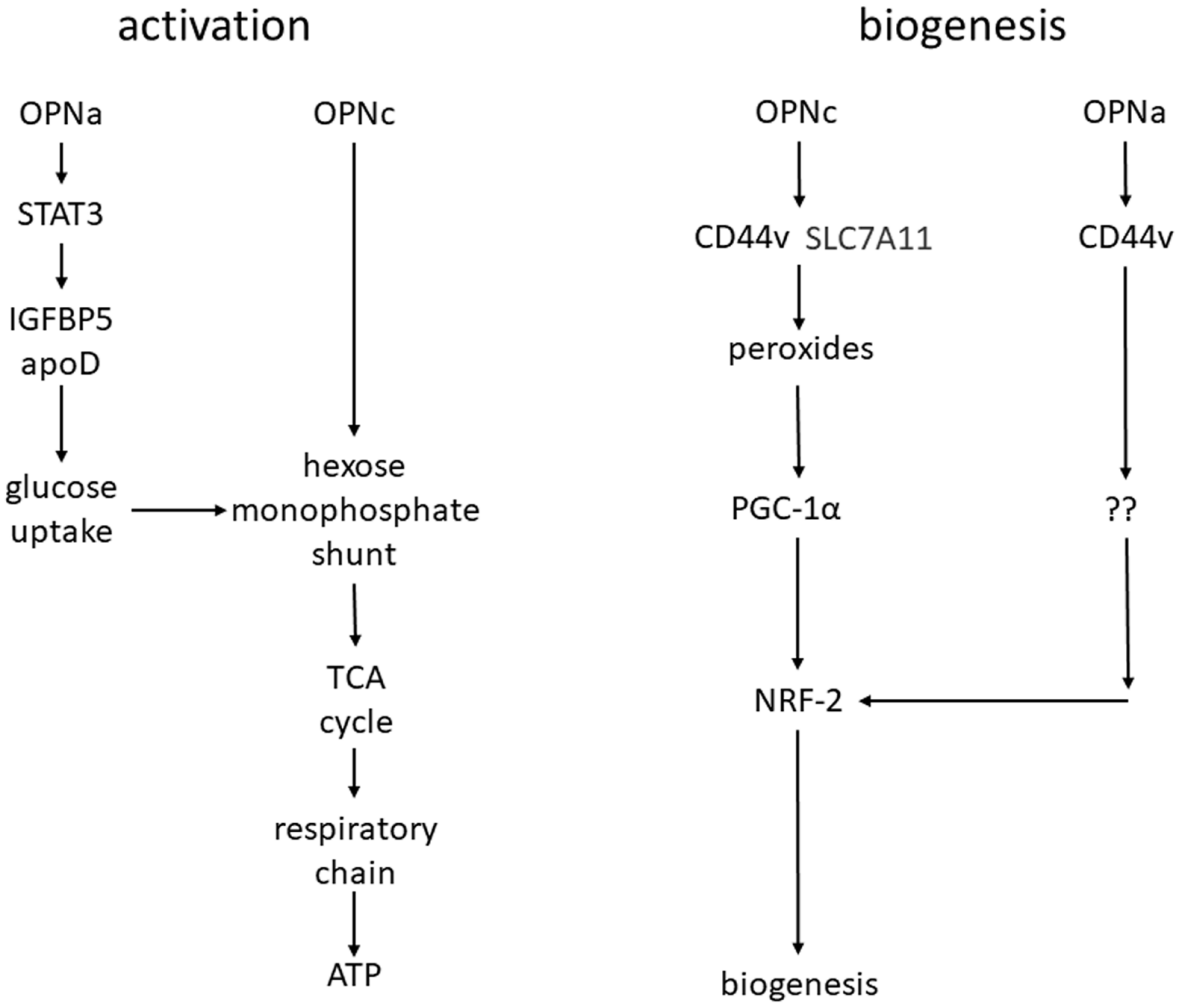
Model for mitochondrial activation and biogenesis. Immediately after deadhesion, mitochondrial energy production is enhanced, with OPNa increasing the cellular glucose uptake and OPNc utilizing this glucose for mitochondrial ATP production (left). Over extended timeframes of deadhesion, the elevated energy requirement is met through mitochondrial biogenesis. OPNc ligates CD44v and SLC7A11 to generate peroxides, induce PGC-1 activity and increase mitochondrial size. OPNa only ligates CD44 (not SLC7A11) and exerts a minor effect on mitochondrial biogenesis (right). The probes employed are shown in Supplementary Figure 6.

The elevated energy requirement during metastasis is met by oxidative phosphorylation and ATP generation in the short run and by mitochondrial biogenesis over longer time frames. Here, we test the hypothesis that both phenomena are induced by Osteopontin variants. The synergy between Osteopontin splice variants -a and -c in breast cancer supports anchorage-independence via increased ATP synthesis from imported glucose and mitochondrial activation of the oxidative metabolism [1, 7, 11]. We have identified PPARG to be activated by Osteopontin under deadherence and in clinical metastases. These observations suggested that Osteopontin splice variants contribute importantly to two mitochondrial adjustments during metastasis, the short-term activation of oxidative metabolism and the longer-term mitochondrial biogenesis (Figure 6). The Osteopontin-induced functions in mitochondrial activation and biogenesis are consistent with its mitochondria-centered support of the enhanced energy requirement over extended timeframes of deadhesion.

During metastasis, cells increase their individual mitochondrial mass and copy number to upregulate the production of ATP as a response to greater energy expenditure. PPARGC1A is elevated in breast cancer metastases and is upregulated in the metastases from various primary tumors to liver and peritoneum. It acts as a master regulator of mitochondrial biogenesis via NRF transcription factors (NRF1 and NRF2, NRF1 is up regulated in breast cancer metastases and in metastases by various cancers to liver, peritoneum, and adrenals) [21]. The NRF proteins belong to a family of transcription factors that are typically activated in oxidative stress. The expression of NRF target genes, such as metallothionein-1 and -2 (MT1 and MT2) [22], can be a downstream readout for PPARG activity. OPN deficiency leads to a lowered PPARG expression [23]. In the absence of exogenous antioxidants, cancer cells maintain redox homeostasis by expressing endogenous antioxidants, many of which are regulated by the redox-sensitive transcription factor nuclear factor (erythroid-derived 2)-like 2 (NFE2L2, NRF2) and its negative regulator Kelch-like ECH-associated protein 1 (KEAP1) [14, 24, 25]. Like NRF2, the transcription factor BTB and CNC homology 1 (BACH1) belongs to the cap’n’collar (CNC) b-Zip family of proteins that bind to antioxidant response elements (AREs) in response to changes in redox states. BACH1 is believed to displace NRF2 from AREs and to act primarily as a transcriptional repressor for antioxidant genes, including HMOX-1, which encodes the detoxification enzyme heme oxygenase 1 (HO-1), and NQO1, which encodes NAD(P)H dehydrogenase (quinone 1) [14]. Our results have shown an increase in nuclear NRF-2 upon deadhesion, but a frequent elevation of NRF-1 expression in human metastasis. The reasons for the seemingly differential use of NRF transcription factors are unknown.

The role of reactive oxygen intermediates in metastasis has been subject to much debate over recent years. Whereas an abundance of oxidative stress can kill the disseminating cells [26, 27], peroxide at lower levels has been shown to be an essential mediator of deadherent expansion [28–30]. Our own research has repeatedly identified the oxidative metabolism to be a main feature of anchorage independence pertaining to OPN signaling [1, 6, 11] or to metastases in general [12]. These diverse observations in the literature are not conflicting. Rather, they may contribute to explanations of metastatic inefficiency [2, 31]. Cancer cells, having been released from their primary tumor, die from anoikis if they cannot overcome the energy deficit of deadhesion. Peroxides are required in the process of energy upregulation. However, released cells that experience oxidative stress also die. Only the very small subset of released cancer cells that generate suitable amounts of peroxide in the right compartment to elevate ATP synthesis without undergoing damaging oxidative stress can survive in the circulation and have the potential to form distant metastases. Of note, frequently, peroxide generation in cancer spread has been attributed to mitochondrial decoupling or leakiness, in which case it may compromise energy generation and limit cancer dissemination. Our observation that peroxide supports anchorage-independence when it is generated far upstream in signaling may shed light on the context-dependence of peroxide effects in metastasis.

Despite a long history of investigation, metastasis remains the least understood aspect of cancer. In 1889, Stephen Paget recognized the non-random distribution of disseminated foci, based on a large number of autopsy records from breast cancer patients. In proposing his seed and soil theory, he suggested that tumor cells resemble seeds that grow out when falling on fertile soil (the specific target organs) – a metaphor with little explanatory content. In 1929, James Ewing observed that metastatic dissemination frequently occurs in organs, which represent downstream connections in the flow of the vascular system. This implied a passive transport of the cancer cells to their target sites (possibly via trapping in the capillary beds). However, in the 1970s, Isaiah Fidler demonstrated that serial passage of cancer cells through mice (injection, extraction of metastases, culture, and reinjection) led to the selection of increasingly aggressive cells. An intrinsic component to metastatic potential had been documented [32]. Yet, individual gene products that can positively or negatively affect metastasis were described only in the late 1980s/early 1990s. Among them was the cell surface receptor CD44v [33, 34]. Through the 1990s, the contributions of these players to migration and invasion became the main focus of study. With the advent of new technical capabilities (*in vivo* microscopy, *ex vivo* PCR) around the turn of the century, there was a recognition that most cancer cells, after having been shed by the primary tumor, die from anoikis in the circulation (the phenomenon of metastatic inefficiency had been observed before [35]), and anchorage-independence moved to the center of attention in efforts to understand the spread of cancer. The metabolic adjustments that achieve deadherent survival and expansion are very different from the Warburg effect. Their characteristics are in the process of being elucidated. The present study seeks to contribute to this goal.

## MATERIALS AND METHODS

### Cells

MCF-7 are breast tumor cells that originated from pleural effusion of a ductal adenocarcinoma. They display a proliferative response to estrogens but grow non-invasively in murine models. They produce no endogenous Osteopontin. MCF-7 stable transfectants of Osteopontin-a (OPNa), Osteopontin-c (OPNc) or vector control have been previously described [6]. MDA-MB-435 cells are of much debated lineage origin [36, 37], but they are cancerous and constitute strong and reliable producers of Osteopontin-a, Osteopontin-b and Osteopontin-c.

### Materials and teagents

The mitochondrial dye MitoTracker^™^ Green FM and the indicator for intracellular reactive oxygen species di(acetoxymethyl ester)(6-carboxy-2’,7’-dichlorodihydrofluorescein diacetate) (DCFH-DA) were bought from Thermo Fisher Scientific. The anti-pan-Osteopontin antibody O-17 came from Assay Designs Inc., the anti-CD44 antibodies (CM0071, CM0241, CM5911) were provided by ECM Biosciences, the anti-Integrin α_v_ β_3_ antibody was obtained from R&D Systems. Antibodies to NRF1 and NRF2 were purchased from Invitrogen and Boster Biological Technology, respectively. The anti-SLC7A11 antibody PA1-16775 (recognizes the N-terminus) was bought from Thermo Fisher Scientific. The PGC-1α inhibitor SR-18292 and activator ZLN005, as well as the SLC7A11 inhibitor HG106 were acquired from Selleckchem. Noble Agar and the SLC7A11 inhibitor erastin2 came from Sigma-Aldrich. The Nunc Glass Base Dish, 12 mm, purchased from Thermo Fisher Scientific, was used in fluorescence microscopy.

### Deadherent growth on poly-HEMA

The indicated cells were seeded at a density of 300,000 cells/well in 6-well plates, either uncoated or in poly (2-hydroxyethyl methacrylate) (poly-HEMA) coated wells (0.12 mg/ml of poly-HEMA in 95% ethanol, allowed to dry overnight, then washed twice with PBS followed by one wash with medium before the initiation of the experiment). Where indicated, the PGC-1α inhibitor SR-18292 was added at the specified concentrations for an incubation time of 24 hours. At the 24-hour timepoint, the cells were lysed for Western blotting.

### Soft agar colony formation

The ability of cells to form colonies in soft agar correlates well with their ability to grow invasively *in vivo*. Colony formation does not only depend on the growth rate of the cells, but also on the expression of gene products for invasiveness and anti-anoikis. We have reported [38] that benign tumor cells do not form colonies in this assay. In contrast, invasive cancer cells form soft agar colonies. The experiment was performed in a 60-mm dish with a bottom layer of 0.5% agar in the culture medium designated to the cells. The bottom agar was allowed to solidify. Cells were then suspended in 0.2% agar (in culture medium) and plated (1 × 10^5^ cells/dish) over the bottom layer. 400 μl culture medium with or without the indicated doses of drug treatment was added every other day for 11 days. The colonies in five microscopic fields (front, back, left, right and center) of each dish were photographed. Colony sizes were measured with Image J (in MCF-7, there were no significant changes in colony frequency [6]; hence there was no need to count cluster numbers). Where indicated, sterile and azide-free monoclonal antibodies were added at 2 μg/plate on day 0, followed by 0.5 μg/plate every other day [39] or polyclonal antibodies were added at 360-fold dilution on day 0 and 666-fold dilution every other day.

### Confocal microscopy and mitochondrial size assessment

Cells were grown in soft agar for 5 days. For analysis, prewarmed (37°C) staining solution containing 100 nM MitoTracker probe was added followed by incubation for 30 minutes. The top agar was removed and placed into Nunc Glass Base dishes for observation under a fluorescence microscope (Leica Stellaris 8 Confocal). The fluorescent images were photographed and then their areas were measured with Image-J.

### Confocal microscopy and assessment of reactive oxygen species

Cells were grown in soft agar for 16 hours. They were then incubated with 5 μM DCFH-DA for 30 mins. For analysis, we separated the top agar from the bottom agar and placed it into Nunc Glass Base dishes, before the cellular fluorescence intensity was visualized with confocal microscopy, photographed and then measured with Image-J as brightness of the proper area in the picture taken. The readouts are relative units, which were converted to set untreated to 100%.

### Immuno-blotting

For the analysis of secreted Osteopontin, serum-free cell culture supernatant was collected from each transfectant. 40 μl of supernatant per sample were electrophoresed on 10% SDS-polyacrylamide mini-gels with non-reducing sample buffer. For the analysis of intracellular Osteopontin, the cells were lysed in RIPA buffer (50 mM Tris-HCl pH 7.5, 150 mM NaCl, 1% NP-40, 0.5% sodium deoxycholate, 0.1% sodium dodecyl sulfate). Cell lysates at equal amounts of protein (10 μg/lane) were electrophoresed on reducing 10% SDS-polyacrylamide gels. The separated proteins were transferred to PVDF membranes and probed with antibody O-17 to pan-Osteopontin as well as antibodies to PGC-1, NRF1, NRF2, Lamin B and α-Tubulin or antibodies to SLC7A11, STAT3 and β-Actin. The expression levels of all transfected gene products were confirmed for every batch within days after thawing and initiation of culture.

### *In vivo* experiments

Balb^nu/nu^ mice received i.p., injection of 1 × 10^6^ MDA-MB-435 cells. Drug treatment was administered i.p., every other day (doses: NAC 100 mg/kg, SR-18292 45 μg/kg) in a total volume of 200 μl PBS. Antibody treatment was given every 4 days at 60 μg in 200 μl PBS. The experiment was terminated on day 21 for necropsy and analysis of tumor spread in the abdominal cavity.

### Statistics

We used ANOVA for the statistical evaluation of the experiments. The results are displayed as mean ± standard error of three independent experiments. Significance was accepted at *p* < 0.05, as indicated in the Figures.

## SUPPLEMENTARY MATERIALS


