# Supplementary material for: Osteopontin induces mitochondrial biogenesis in deadherent cancer cells
Source: Oncotarget. 2023 Dec 1;14:957–69. doi: 10.18632/oncotarget.28540 (PMC10691814; doi:10.18632/oncotarget.28540)
Supplement: Supplementary file 1 [file oncotarget-14-28540-s001.pdf]

Osteopontin induces mitochondrial biogenesis in deadherent cancer cells

SUPPLEMENTARY MATERIALS

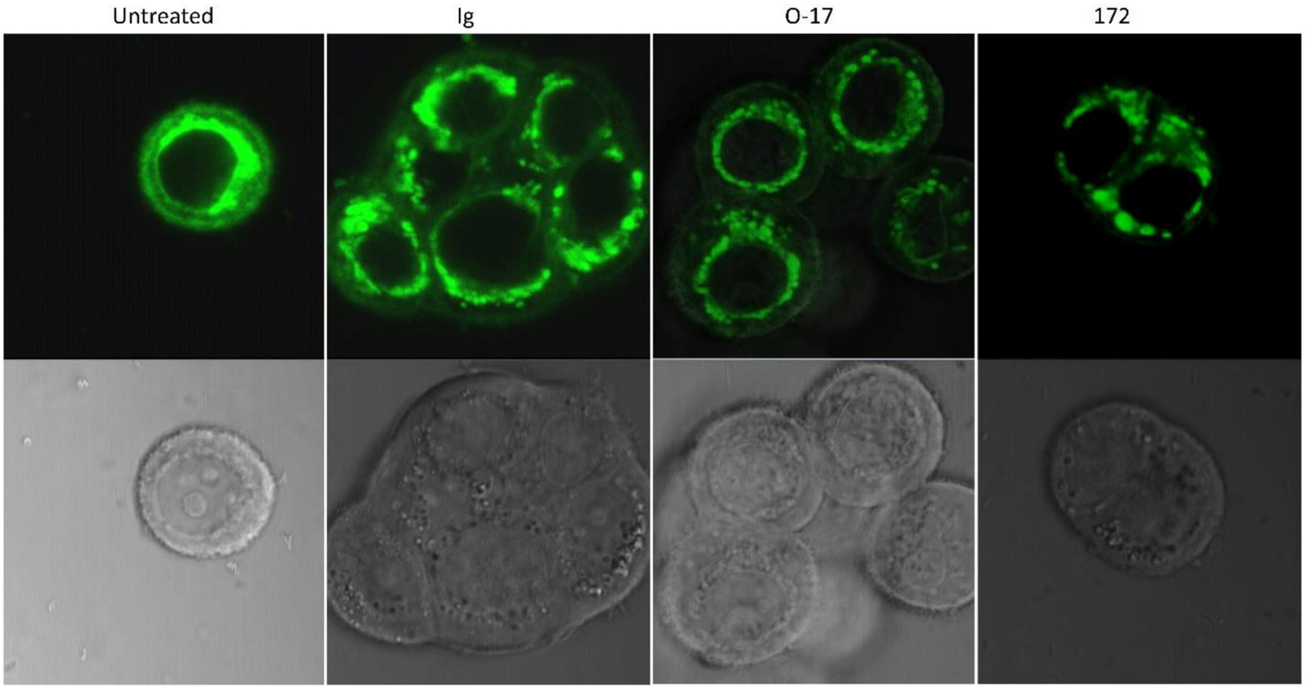

Supplementary Figure 1: Biogenesis mediated by endogenous OPN. MDA-MB-435 cells were plated in soft agar. Every other day, they were supplemented with medium or treated with control Ig, anti-OPN antibody O-17, anti-OPNc antibody clone 172. On day 5, ensuing to staining with a mitochondrial dye, fluorescence microscopy pictures (top row, with brightfield comparison in the bottom row) were taken

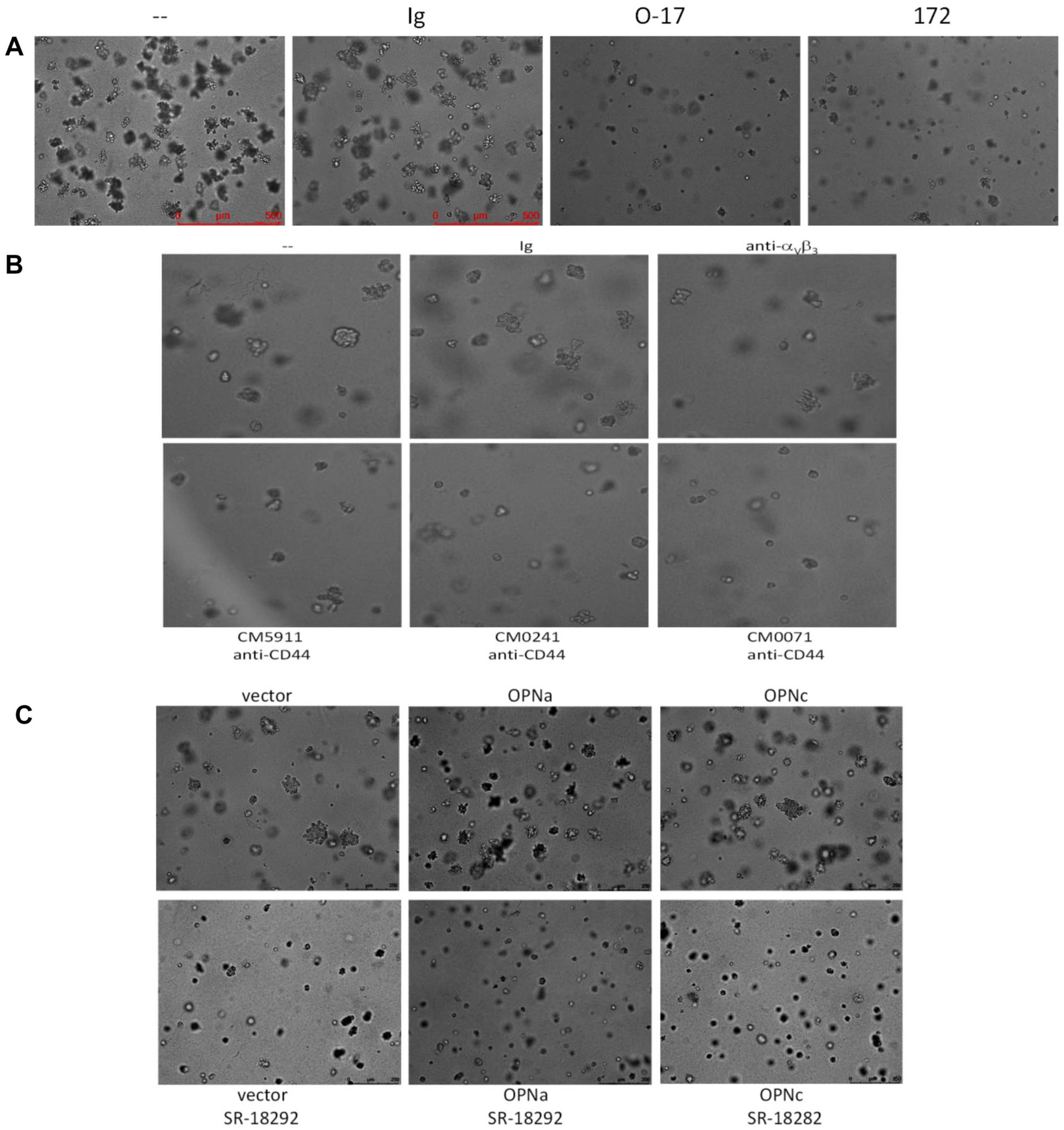

Supplementary Figure 2: Microscopy of soft agar colonies. (**A**) Antibody inhibition of soft agar colony formation by MDA-MB-435 cells. MDA-MB-435 cells were plated in soft agar. Every other day, they were treated with anti-OPN antibody O-17, anti-OPNc antibody clone 172, control Ig or left untreated. The colony sizes were photographed after 14 days. (**B**) Receptor usage by OPN as assessed with antibodies to the CD44 variable domain, to the CD44 N-terminus or to Integrin $\alpha_V\beta_3$. Shown are the results from MCF-7 OPNa cells (**C**) PGC-1α signaling in soft agar colony formation. Measurements of colony formation by MCF-7 transfectants in the presence or absence of the PGC-1α inhibitor SR-18292.

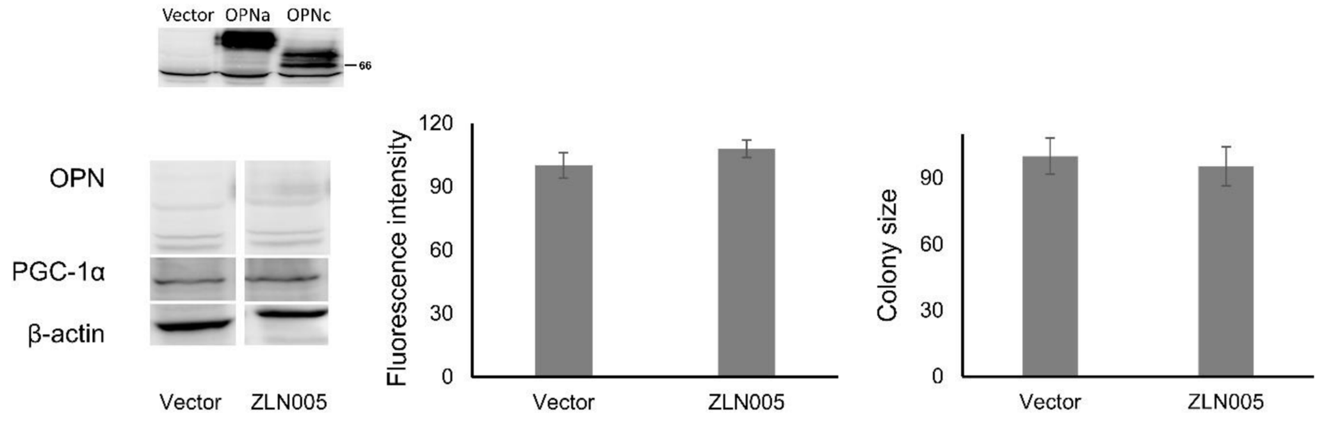

Supplementary Figure 3: Lack of efficacy by the PGC-1 activator ZLN005. The upper left panel shows the OPN expression by MCF-7 transfectants according to Western blotting of serum-free cell culture supernatant. MCF-7 vector cells were treated with PGC-1α activator ZLN005. Despite being a transcriptional inducer, ZLN005 did not alter PGC-1α expression. The bar graphs show mitochondria size (middle) and colony size (right). The untreated controls are the same as shown in Figure 2.

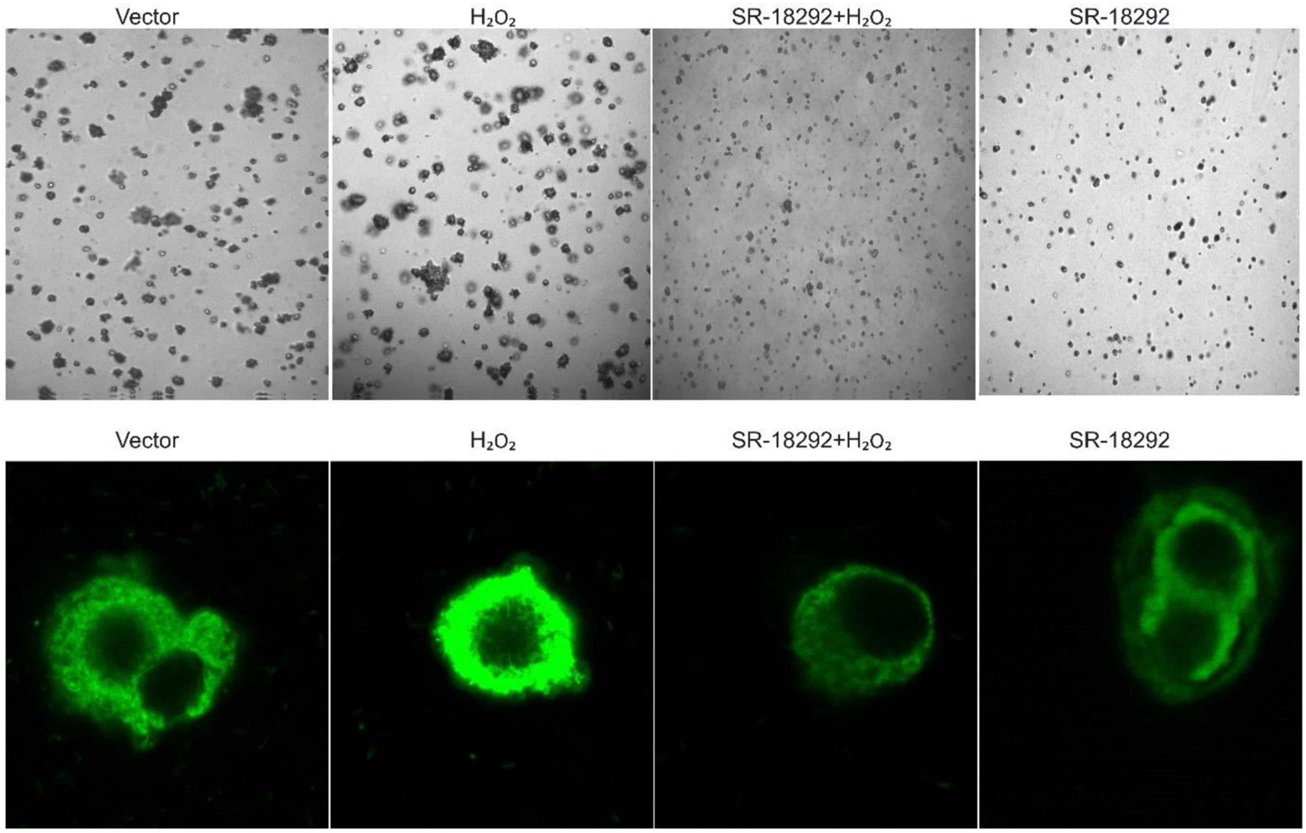

Supplementary Figure 4: H_2O_2 and PGC-1 signaling. Representative photos for treatment of deadherent MCF-7 vector cells with 10 µM H_2O_2, 10 µM of the PGC-1α inhibitor SR-18292, or both before assessment of colony size (upper panel, 11 days) and mitochondria size (lower panel, 5 days).

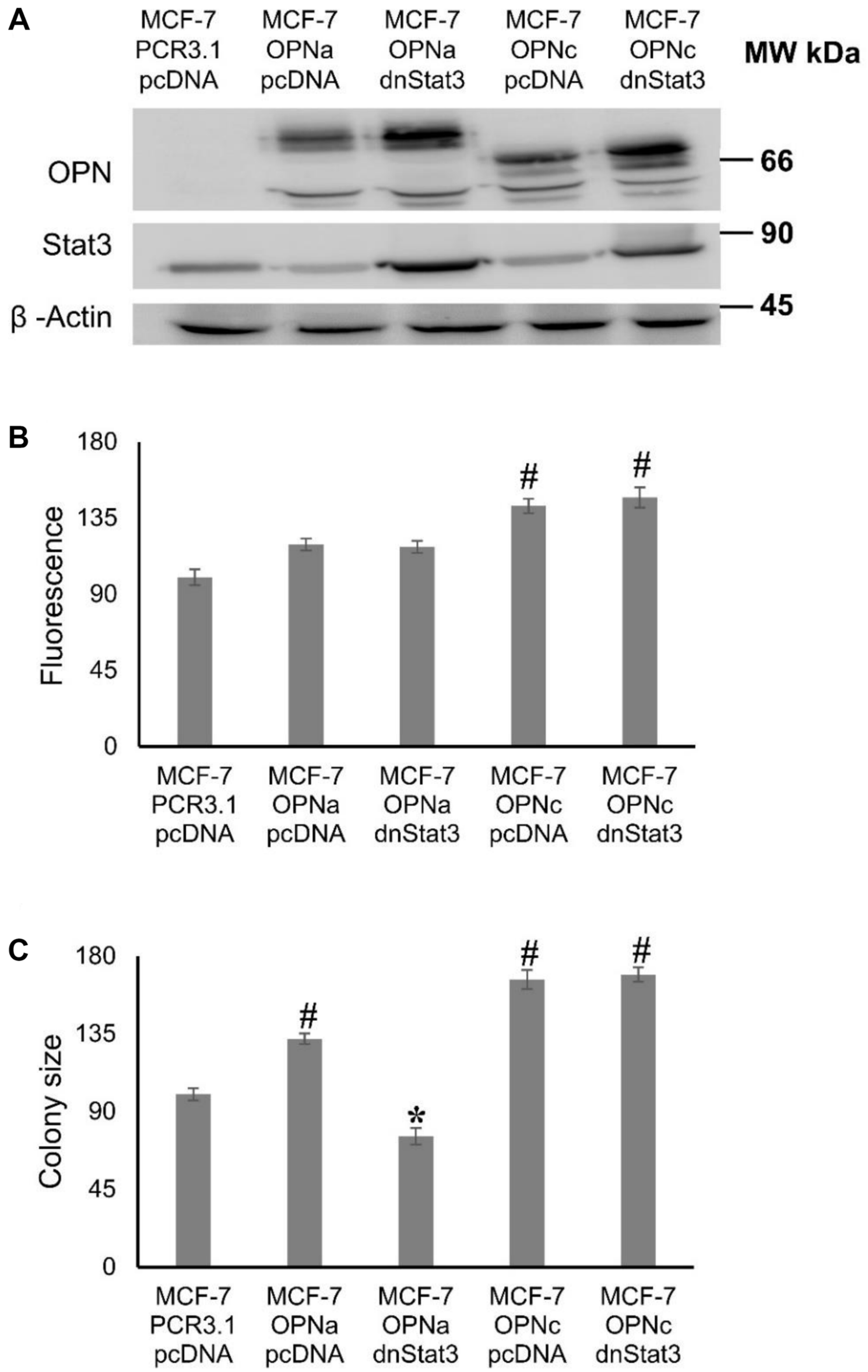

Supplementary Figure 5: STAT3 as a downstream target for OPNa signaling is not involved in mitochondrial biogenesis. (**A**) Western blot for confirming the expression of OPN and STAT3 in transfected MCF-7 cells. β-Actin served as a loading control. (**B**) Lack of effect by dnSTAT3 on the size of mitochondria. (**C**) Selective reduction by dnSTAT3 of the soft agar colony formation by OPNa but not by OPNc. Significance was assessed with the one-tailed *T*-test for paired samples.

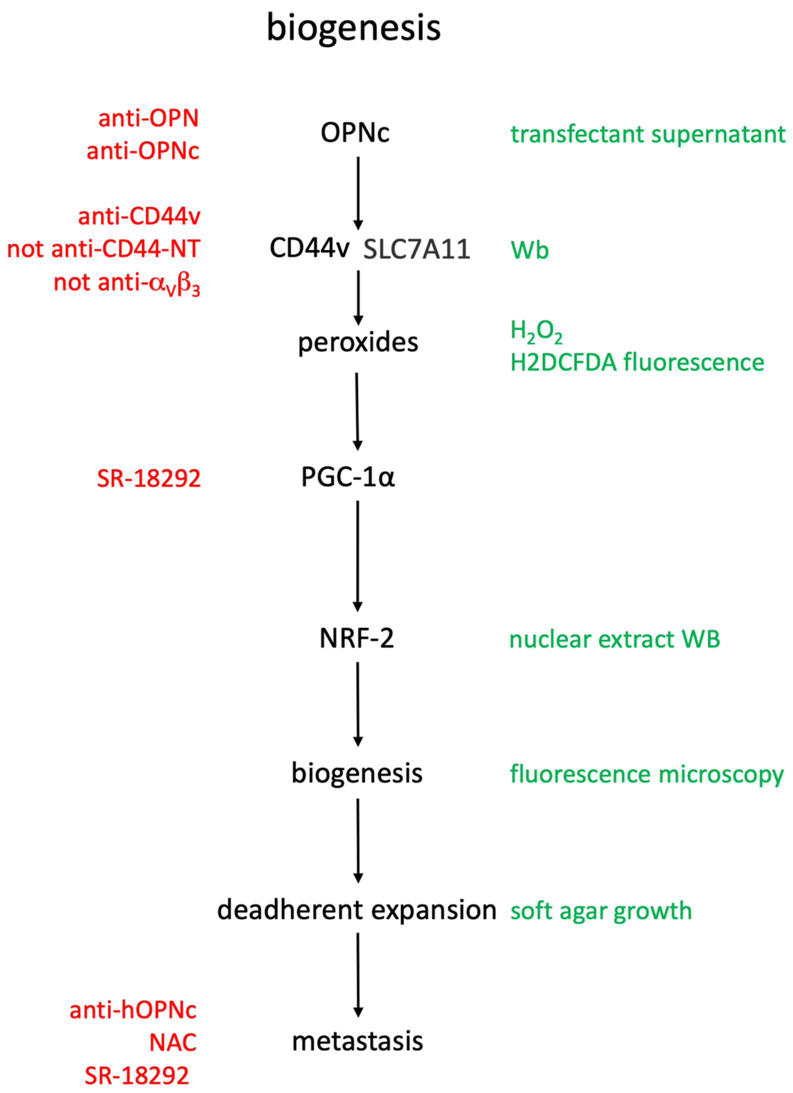

Supplementary Figure 6: Evidence for the pathway of mitochondrial biogenesis. Over extended timeframes of deadhesion, the increased energy requirement is met through mitochondrial biogenesis. OPNc ligates CD44v and SLC7A11 to generate peroxides, induce PGC-1 activity and increase mitochondrial size. Activators and direct evidence are shown in green on the right. Inhibitor probes are shown in red on the left.
